# Biomaterials barcoding: a high-throughput breakthrough

**DOI:** 10.1186/s43556-023-00163-x

**Published:** 2024-01-02

**Authors:** Masoud Mozafari

**Affiliations:** Research Unit of Health Sciences and Technology, Faculty of Medicine, University of Oulu, Oulu, Finland

## Abstract

In the world of biomedical breakthroughs, Rice University bioengineer Omid Veiseh and his team are making waves with their recent publication in Nature Biomedical Engineering (2023) (Mukherjeeet al., Nat Biomed Eng. 7:867–886, 2023). This study is a pivotal step in our fight against fibrosis, an issue that has long hindered medical progress. Their pioneering research isn’t just a scientific milestone; it’s a game-changer in how we tackle tissue scarring. Veiseh and his team have introduced an innovative method that allows for rapid testing of various materials within living organisms. By employing cellular barcoding and cutting-edge sequencing techniques, they’ve accelerated the assessment of multiple hydrogels. As we delve deeper into the specifics of this groundbreaking study, we uncover not just scientific insights, but the potential to revolutionize how we conceptualize and utilize biomaterials. This discussion isn’t merely about research methods; it’s about the ray of hope and boundless opportunities this study illuminates across the spectrum of biomaterials science.

Implanted devices often trigger a complex host immune response, leading to inflammation, cellular deposition, and ultimately fibrotic overgrowth. This process is a significant hurdle in the development of biomaterials with improved biocompatibility. Among the extensively studied biomaterials, alginate-based hydrogels have found applications in various fields. Alginate-based hydrogels are a well-established biomaterial due to their biocompatibility, mild gelation conditions, and ease of modification, making them attractive for various biomedical applications [1]. However, these hydrogels also face host immune recognition challenges, especially when encapsulating donor tissue [2]. To address this challenge, various approaches, such as chemical modification and adjustments to implant geometry, have been explored [3]. The study by Mukherjee et al. represents a significant step forward in tackling this issue through high-throughput screening.

Traditional in vivo biomaterial screening methods have limitations, often involving one animal or implantation site for a single biomaterial. Mukherjee and colleagues propose a novel approach that employs cellular barcoding to screen multiple hydrogel formulations within a single host. This approach eliminates the need for an extensive number of animals and substantially increases the throughput of biomaterial screening. To achieve this, they encapsulate distinct barcoding cells within hydrogel capsules made from new alginate analogues. These barcoding cells, each representing a unique biomaterial, are human umbilical vein endothelial cells (HUVECs) with distinct genetic profiles, identified through single nucleotide polymorphisms (SNPs) via next-generation sequencing. The cellular barcoding technique enables the researchers to screen up to 20 distinct biomaterials in a single mouse and over 100 biomaterials in a non-human primate, representing a remarkable increase in throughput. Moreover, a dual-donor barcoding strategy was introduced to expand the library to 400 different codes, allowing even greater biomaterial screening throughput. This innovative approach has the potential to revolutionize the way we identify biomaterials with antifibrotic properties, streamlining the process and minimizing the number of living subjects required [4]. This strategy represents a pivotal step forward in research methodologies, aligning scientific progress with ethical considerations by ensuring rigorous testing while minimizing the impact on animal subjects.

The study not only demonstrates the effectiveness of this high-throughput screening method but also showcases the practical applications of the identified lead hydrogel formulations (Fig. 1). One of the lead formulations, Z4-A10, was applied to encapsulate xenogeneic human pancreatic islets, resulting in long-term glycaemic control in a pro-fibrotic mouse model. This breakthrough has promising implications for the field of diabetes therapy, offering an alternative to immunosuppressive drugs. Additionally, two other lead hydrogels, Z1-A3 and B2-A17, were used to coat medical-grade catheters, preventing fibrotic overgrowth and enhancing their biocompatibility. This work highlights the potential of these hydrogels for improving the long-term performance of medical devices and therapeutic cell encapsulation. The study delves further into the structural features of the lead small molecules, shedding light on commonalities among the top-performing hydrogels. Hydrophilic PEG linkers, a hydrophilic surface, and the presence of certain structural motifs were found to be associated with improved antifibrotic properties. This structural insight offers guidance for future material design, potentially opening avenues for the development of new antifibrotic hydrogel formulations [6].

**Fig. 1:**
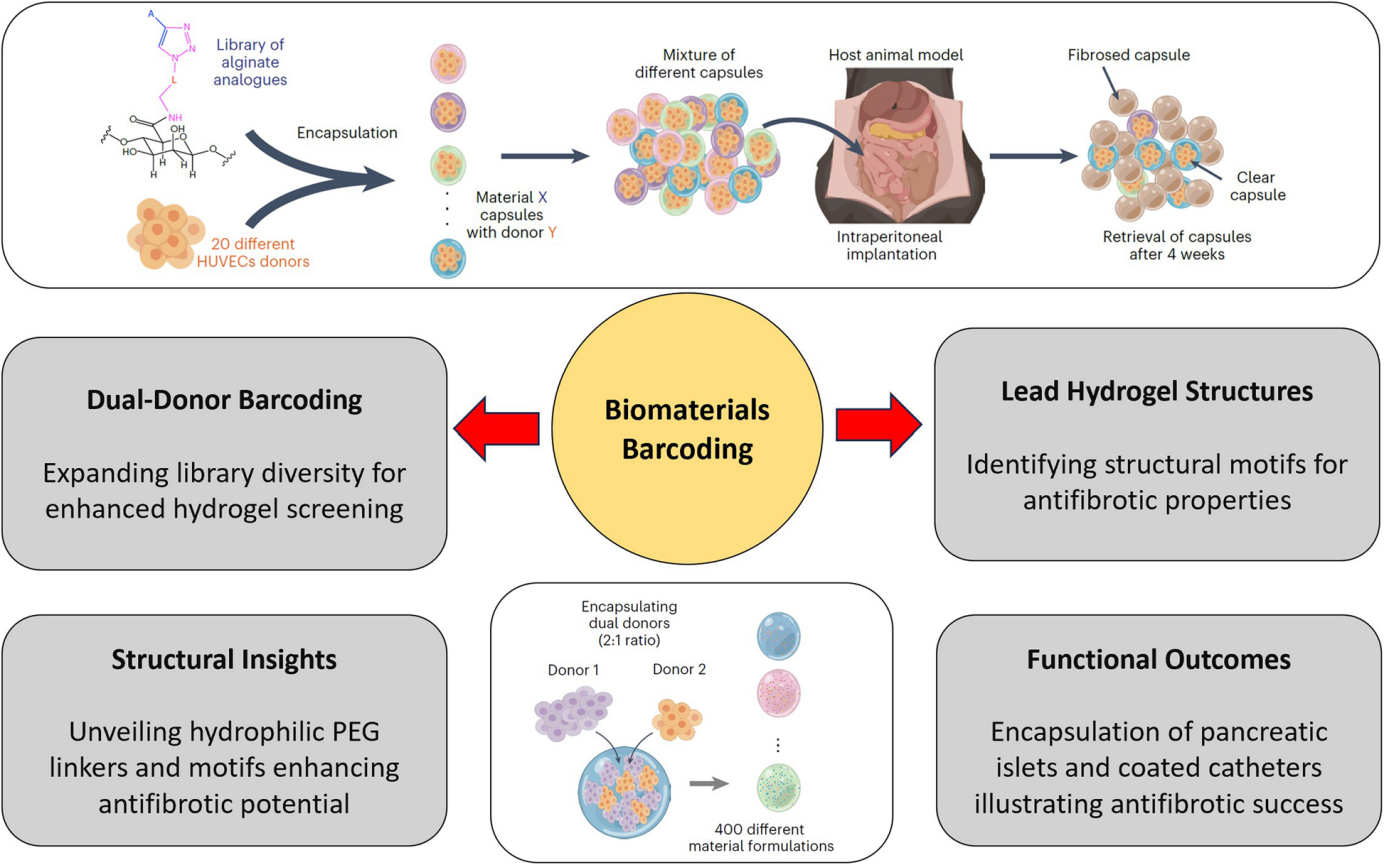
High-Throughput Biomaterial Screening Enabled by Cell Barcoding. It is showcasing the innovative high-throughput in vivo screening method employing cellular barcoding and next-generation sequencing. The figure depicts the barcoding technique, exhibiting the encapsulation of distinct barcoding cells within hydrogel capsules in mice and non-human primates. It visualizes the high throughput of material screening, the structural insights into lead hydrogels, functional outcomes in encapsulating pancreatic islets, and coated catheters, emphasizing their potential applications. The figure also hints at future directions in refining species distinction, exploring long-term stability, toxicity, and advancing biomaterial screening methods. (Reprinted with permission from [5])

While the study represents a significant advancement in biomaterial screening, it also has its limitations. Cross-species genetic homology between humans and primates can pose challenges in differentiating genetic signals, and additional work is required to refine species distinction. Furthermore, enhancing DNA output from highly fibrosed capsules is essential for the comprehensive identification of biomaterials. The long-term stability, toxicity, and biocompatibility of lead small molecules also need further investigation [7]. In evaluating the biomaterials barcoding technology, it’s pivotal to assess its feasibility, reliability, safety, and economic implications to understand its practicality and widespread implementation. The feasibility of this high-throughput platform lies in its ability to efficiently screen numerous biomaterials within a single living subject, significantly reducing the number of required animals and time. Moreover, ensuring the reliability of barcoding techniques and sequencing methodologies is crucial to ascertain accurate identification and characterization of biomaterials. Safety considerations encompass the host response to the barcoded biomaterials, emphasizing biocompatibility and the absence of adverse effects, ensuring the translated clinical applicability. Addressing the economic aspects involves assessing the cost-effectiveness and scalability of the technology, considering the expenses associated with next-gen sequencing, cell barcoding, and data analysis. Comprehensive evaluation across these domains will provide an encompassing understanding of the viability and potential challenges of implementing biomaterials barcoding platforms in biomedical research and clinical applications.

In summary, Mukherjee et al.’s pioneering study marks a new era in the field of biomaterials research, particularly in combating fibrosis. By introducing a high-throughput in vivo screening method using cellular barcoding and next-generation sequencing, they have not only expanded the throughput of material screening but have also identified promising lead hydrogel formulations with antifibrotic properties. These lead hydrogels hold significant promise in improving the biocompatibility and long-term performance of medical devices and therapeutic cell encapsulation. With potential applications in diabetes therapy and medical device coatings, this research has the potential to benefit millions of patients worldwide. It exemplifies the power of innovative thinking and interdisciplinary collaboration in addressing complex biomedical challenges.

## Data Availability

Not applicable.
